# Influence of the Virus LbFV and of *Wolbachia* in a Host-Parasitoid Interaction

**DOI:** 10.1371/journal.pone.0035081

**Published:** 2012-04-25

**Authors:** Julien Martinez, Anne Duplouy, Megan Woolfit, Fabrice Vavre, Scott L. O'Neill, Julien Varaldi

**Affiliations:** 1 CNRS UMR5558 Laboratoire de Biométrie et Biologie Evolutive, Université Lyon 1, Villeurbanne, France; 2 Department of Biological and Environmental Sciences, University of Helsinki, Helsinki, Finland; 3 School of Biological Sciences, Monash University, Clayton, Victoria, Australia; University of Poitiers, France

## Abstract

Symbionts are widespread and might have a substantial effect on the outcome of interactions between species, such as in host-parasitoid systems. Here, we studied the effects of symbionts on the outcome of host-parasitoid interactions in a four-partner system, consisting of the parasitoid wasp *Leptopilina boulardi*, its two hosts *Drosophila melanogaster* and *D. simulans*, the wasp virus LbFV, and the endosymbiotic bacterium *Wolbachia*. The virus is known to manipulate the superparasitism behavior of the parasitoid whereas some *Wolbachia* strains can reproductively manipulate and/or confer pathogen protection to *Drosophila* hosts. We used two nuclear backgrounds for both *Drosophila* species, infected with or cured of their respective *Wolbachia* strains, and offered them to *L. boulardi* of one nuclear background, either infected or uninfected by the virus. The main defence mechanism against parasitoids, i.e. encapsulation, and other important traits of the interaction were measured. The results showed that virus-infected parasitoids are less frequently encapsulated than uninfected ones. Further experiments showed that this viral effect involved both a direct protective effect against encapsulation and an indirect effect of superparasitism. Additionally, the *Wolbachia* strain *w*Au affected the encapsulation ability of its *Drosophila* host but the direction of this effect was strongly dependent on the presence/absence of LbFV. Our results confirmed the importance of heritable symbionts in the outcome of antagonistic interactions.

## Introduction

Endosymbionts are extremely frequent in arthropods, especially in insects. By providing additional heritable genetic material, they may contribute to the adaptation of their insect host [1], [2]. A growing literature reports examples of beneficial effects provided by heritable endosymbionts to their hosts when the latter are engaged in antagonistic relationships with other species [3], [4], [5], [6], [7]. Host-parasitoid systems are therefore of great interest, as both protagonists may harbour symbiotic organisms influencing the outcome of their interaction, thus offering additional routes towards resistance or virulence besides host nuclear factors [8]. Indeed, it has been found that several bacteria protect their insect host from parasitoid-induced mortality in aphids [9], [10], [11] or in *Drosophila hydei* [12]. On the other hand, many insect parasitoids harbour viral symbionts allowing them to cope with the host's immune defenses, increasing the virulence of these parasitoids [13], [14], [15], [16]. These heritable viruses are injected into the parasitoid's host together with the eggs, suppressing, to varying degrees, the immune reaction of the parasitized host [17].

Whereas most studies have focused on the effect of a single symbiont either in the host or in the parasitoid on the outcome of the host-parasitoid interaction (but see [18]), we have investigated here the potential influence of two different symbionts, one infecting the host and the other infecting the parasitoid. This system involves the parasitoid wasp *Leptopilina boulardi* that is able to parasitize both *Drosophila melanogaster* and *D. simulans* larvae. Parasitization may lead to three different outcomes: (i) the parasitoid avoids the immune system of the *Drosophila* larva, reaches the adult stage and ultimately kills the *Drosophila*; (ii) the *Drosophila* succeeds in killing the parasitoid by a cascade of immune reactions leading to the encapsulation of the young wasp [19]; (iii) the interaction ends with the death of both protagonists.

*Drosophila* species are often infected by the maternally-transmitted bacterium *Wolbachia*. Different strains have been described, some inducing various reproductive manipulations in their hosts, such as cytoplasmic incompatibility, while others have unknown effect. This raises the question of the mechanism explaining their prevalence in natural populations [20], [21]. One hypothesis is that non-manipulating *Wolbachia* strains may increase the resistance of their *Drosophila* host to parasitoid attacks. It has been found that *Wolbachia* can confer resistance against various parasites such as RNA viruses [6], [22], [23], [24], [25], filarial nematodes and *Plasmodium* [26], [27], [28], [29]. Moreover, manipulating strains could combine the advantage of both a reproductive manipulation and a protective effect, improving even more their invasive potential. Counter-examples contrasting with protective effects found against pathogens were however also previously described. *Wolbachia*-infected *D. simulans* have, for instance, reduced ability to encapsulate parasitoids [18]. Similarly, *Wolbachia* in the isopod *Armidillidium vulgare* is able to infect host haemocytes [30], decreasing the immune-competence of its host, particularly by affecting the prophenoloxidase activity [31], a key pathway of the immune system in arthropods [32].

The parasitoid *L. boulardi* is often infected by a maternally-transmitted DNA virus called LbFV (*Leptopilina boulardi* Filamentous Virus), whose prevalence may exceed 90% in some locations [33]. This virus manipulates the behavior of adult females in a way that favours its own transmission [34]. Whereas virus-free females lay a single egg in encountered *Drosophila* larvae and usually avoid superparasitism, i.e. laying eggs in already parasitized larvae, virus-infected females readily lay eggs in previously parasitized host larvae. Infected offspring are consequently exposed to strong competition, as only one parasitoid is able to fully develop inside a single host larva. Superparasitism is adaptive for the virus as it enables its horizontal transmission among the parasitoid larvae competing within the same fly larva. Theoretical work has shown that the virus is selected for increasing the natural superparasitism tendency of the parasitoid because it allows infection of new parasitoid matrilines [35]. Additionally, both the vertical and the horizontal transmission of the virus may be facilitated by increased virulence of the parasitoid against *Drosophila*'s immune response.

In this paper, we tested the combined effect of LbFV (infecting the parasitoid) and of different strains of *Wolbachia* (infecting the *Drosophila* host) on the outcome of the host-parasitoid interaction using two genetic backgrounds of *D. melanogaster* and *D. simulans* and one genetic background of *L. boulardi*. We measured the successful encapsulation rate by counting adult flies that survived parasitoid attack. In a second experiment, we also controlled for the occurrence and effect of superparasitism by measuring encapsulation in *Drosophila* larvae. The results showed that symbionts indeed influence the final outcome in this host-parasitoid interaction.

## Methods

### Insect lines and rearing conditions

Two nuclear backgrounds of each of *Drosophila melanogaster* (YW-BNE and *w* ^1118^) and *D. simulans* (CO and DSR) were used, either infected with or cured of different *Wolbachia* strains, leading to eight inbred lines as described in Table 1. All flies were reared under a 12L∶12D photoperiod at 21°C and fed with a standard diet [36]. Cured lines were obtained by mixing in each fly vial, 0.5 mL of a 100 µg/mL rifampicin antibiotic solution to the 10 mL/vial fly food, for three generations. To eliminate any potential direct effect of the antibiotics, *Drosophila* lines were then reared on antibiotic-free food for several generations before the start of the experiments. Their *Wolbachia* infection status was checked by PCR detection using the 81F-691R *wsp* primers specific to *Wolbachia* [37].

**Table 1:** Description of the *Drosophila* lines with their respective *Wolbachia* strain, and the *L. boulardi* lines.

Insect species	Nuclear background	Origin	Symbiont strain	Reference
*D. melanogaster*	YW-BNE	Toowong, Brisbane, Australia	*w*Mel	[55]
			Cured	This study
	*w* ^1118^	Pasadena, California, USA	*w*MelPop	[56]
			Cured	This study
*D. simulans*	CO	Coffs Harbour, Australia	*w*Au	[20]
			Cured	This study
	DSR	Riverside, California, USA	*w*Ri	[57]
			Cured	This study
*L. boulardi*	Sienna9	Sienna, Italy	LbFV particles from a French population	[38]
			Uninfected	[38]

Two reference lines of *L. boulardi*, designated NSref and Sref, with the same nuclear genetic background but a different virus-infection status were used (Table 1). NSref is an inbred uninfected line (with an estimated homozygosity greater than 82%) originating from Sienna (Italy), that lay only one egg per *Drosophila* larva on average [38]. Sref is LbFV-infected and is derived from the NSref line, which was infected with viral particles originating from the south of France (Gotheron) via natural horizontal transfer, after a superparasitism event. This newly infected line proved stable over generations for virus infection and susceptible to the behavioral manipulation exerted by LbFV (increase in superparasitism tendency). Before the start of our experiments, parasitoids were maintained under a 12L∶12D photoperiod at 26°C, on a laboratory *Wolbachia*-free *D.melanogaster* line originating from Lyon (France). Both NSref and Sref have been shown to be *Wolbachia*-free in a previous study [38]. Viral infection status of these two *L. boulardi* lines was determined by diagnostic PCR using the primers 500-R/102F designed for specific detection of LbFV [39]. LbFV has, to date, never been found in *Drosophila* hosts [39].

### Experiment 1: Contribution of LbFV and Wolbachia to the host-parasitoid interaction

In experiment 1, we addressed the question of the contribution of LbFV and *Wolbachia* on several key traits of the *Drosophila*-parasitoid interaction. For each line, one hundred eggs were deposited into rearing vials (n = 40 per *Drosophila* nuclear background per *Wolbachia* infection status combination, 320 vials in total). Twenty-four hours later, a single female parasitoid, either LbFV-infected or not, was introduced into each vial (n = 15 for each parasitoid infection status) and removed 24 hours later. Ten control vials for each *Drosophila* line (*Wolbachia*-infected and *Wolbachia*-free lines) were kept without parasitoid. Experiments were carried out in large incubators at 26°C under 12L∶12D photoperiod and 70% relative humidity.

From day 7, *Drosophila* flies that were not parasitized, or were parasitized but eliminated the parasitoid, started to emerge and were collected daily and counted at the end of the experiment. In response to parasitism, *Drosophila* larvae can initiate a protective immune reaction, which can lead to the encapsulation of the parasitoid egg or larva [19]. Successful encapsulations are easily detected in the adult flies' abdomens, under a stereomicroscope, by crushing the entire individual between two glass slides. The number of flies containing capsules was recorded. Parasitoids started to emerge 12 days after the emergence of the first flies (day 19), were removed from the vials and counted at the end of the experiment. For technical reasons, the experiment was split into two temporal blocks, half of the vials of each treatment being launched on one day and the other half on the following day.

### Fitness-related traits involved in the *Drosophila*-parasitoid interaction

**Figure 1:**
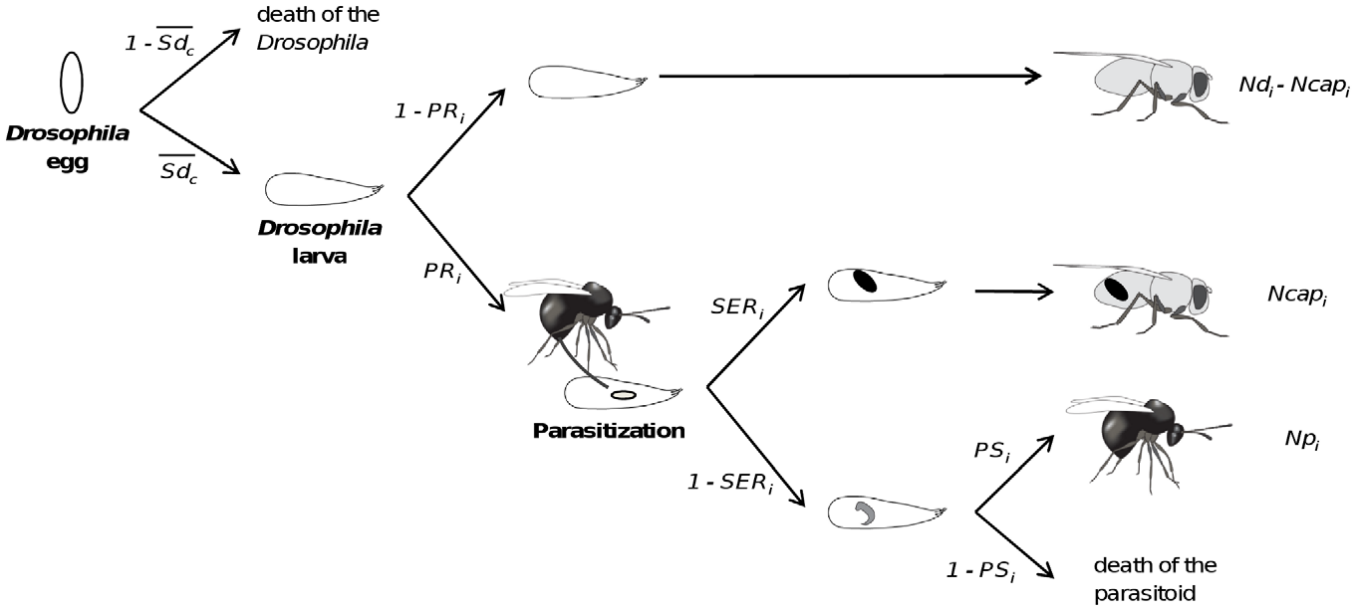
Temporal sequence of the *Drosophila*-parasitoid interaction and key life-history traits. *Drosophila* natural mortality (not due to parasitism) was assumed to occur early, before introduction of the parasitoid. (*Sd_c_*): mean *Drosophila* survival in control vials, (*PR_i_*): parasitism rate in vial *i*, (*SER_i_*): successful encapsulation rate in *i*, (*PS_i_*): parasitoid developmental success in *i*, (*Ncap_i_*): number of flies with successful encapsulation in *i* and (*Np_i_*): number of emerging parasitoids in *i*.

Different key life-history traits influencing the outcome of the *Drosophila*-parasitoid interaction were measured (Figure 1). The parasitism rate (*PR_i_*), or the proportion of *Drosophila* larvae parasitized by a single female parasitoid in a given vial *i*, was estimated by comparing the number of emerged flies in the treatment vial *i* (*Nd_i_*) to the mean number of flies in the control vials (*N_c_*) of each *Drosophila* line as follows:
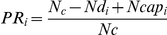
with *Ncap_i_* being the number of adult flies containing capsules in vial *i*.

From this estimator, the successful encapsulation rate (*SER_i_*), defined as the proportion of parasitized *Drosophila* larvae that survived up to the adult stage, was calculated by dividing the number of flies containing capsules by the estimated number of parasitized larvae:
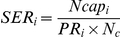
The parasitoid developmental success (*PS_i_*), defined as the proportion of parasitoids that survived up to the adult stage after successfully avoiding encapsulation, was calculated as follows:
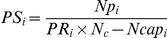
with *Np_i_* being the number of adult parasitoid offspring in vial *i*.

### Overall fitness

We used the number of adult parasitoid offspring *Np_i_* as the best approximation of the female parasitoid's overall fitness. To take into account variation in intrinsic mortality among the *Drosophila* nuclear backgrounds, we calculated an index of the *Drosophila*'s fitness relative to their natural mortality, i.e. in the absence of parasitoid. *Drosophila* fitness (*Sd_rel_*) exposed to parasitoids was thus defined as the fly survival in vial *i* (*Sd_i_*) relative to their respective survival in control vials (*Sd_c_*):
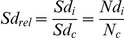



### Experiment 2: Direct and indirect effect of the virus on encapsulation

Virus-infected and uninfected parasitoids display contrasting egg-laying strategies (frequent superparasitism for Sref and rare for NSref). The virus' effects on the outcome of host-parasitoid interactions may therefore either result from a direct effect of the virus or from an indirect effect through the occurrence of superparasitism. In order to distinguish between these effects, we performed a second experiment using only the cured DSR line. We chose this particular line for its successful encapsulation rate, clearly dependent on the parasitoid infection status. Forty vials were prepared for both uninfected and virus-infected parasitoid lines. Half of these contained 100 *Drosophila* eggs deposited on day 1 (experiment 2.1), and the other half contained 125 eggs deposited on day 2 (experiment 2.2). We used two *Drosophila* densities (100 eggs or 125 eggs) in order to vary the host/parasitoid ratio and possibly the frequency of superparasitism. For each larval density, ten additional vials without parasitoid were used as controls. From each treatment vial, ten randomly chosen *Drosophila* pupae were dissected under a stereomicroscope. We recorded the number of parasitoid eggs, parasitoid larvae and the number of capsules found in each pupa. The larval encapsulation rate (*LER_i_*) is the proportion of fly larvae that encapsulated all parasitoids. For this analysis, we only considered fly larvae containing either one or two parasitoids since larvae containing more than two parasitoids were too rare to support strong statistical analyses. The encapsulation rate at adult stage (*SER_i_*) was measured as previously described in this paper except that dissected larvae were taken into account by subtracting 10 flies from the mean number of flies in the control vials (*_Nc_*).

### Statistical analyses

All data sets were analysed with the R software (version 2.11.1) (R Development Core Team, 2005). Except for the larval encapsulation rate, all life-history traits were analysed using linear models after adequate transformation to reach the assumptions of normality and homoscedasticity. Some experimental vials in which parasitoids did not lay any eggs were disregarded from the analyses. Linear models were constructed by putting first the temporal block effect in order to remove the potential stochastic effects before testing the parameters of interest, i.e. the effects of the two symbionts. The larval encapsulation rate was analysed using a generalized linear model with a binomial error structure (logit link function) given the binary nature of the data (encapsulation of all parasitoids or not).

## Results

### Experiment 1: Contribution of LbFV and Wolbachia to the host-parasitoid interaction

In the global analysis of experiment 1, there was a contribution of the temporal block (either on its own or in complex interaction with other factors, Table 2) suggesting that some environmental parameters that were not controlled for in this experiment significantly influenced the outcome of the *Drosophila*-parasitoid interaction. Moreover, the *Drosophila* nuclear background was always significant showing that the outcome of host-parasitoid interaction was highly dependent on the host genotype (Table 2). Particularly, analyses per *Drosophila* nuclear background showed that the complex patterns of statistical interactions observed in the global analysis were mostly due to CO flies (Table S1). In the next sections, we will focus on symbiont effects and their interactions, consistently with our main goal.

**Table 2:** Analysis of variance of life-history traits of the host-parasitoid interaction in experiment 1.

Parameters	df	Successful encapsulation rate (square root-transformed)		Parasitism rate (arcsine square root-transformed)		Parasitoid developmental success (square root-transformed)		Number of parasitoid offspring (square root-transformed)		Drosophila relative survival (log-transformed)	
		*F*	*P*	*F*	*P*	*F*	*P*	*F*	*P*	*F*	*P*
Block (1)	1	11.25	0.001*	2.04	0.15	3.55	0.06	1.74	0.19	10.65	0.001*
*Drosophila* nuclear background (2)	3	4.08	0.008*	5.71	0.0009*	15.89	<0.0001*	36.15	<0.0001*	8.36	<0.0001*
Virus (3)	1	46.55	<0.0001*	3.65	0.06	8.47	0.004*	0.88	0.35	31.93	<0.0001*
*Wolbachia* (4)	1	1.15	0.28	0.0001	0.99	5.99	0.02*	3.73	0.054	0.21	0.65
interactions											
(1)×(2)	3	2.38	0.07	1.05	0.37	1.39	0.25	0.84	0.48	0.31	0.82
(1)×(3)	1	0.2	0.65	4.47	0.04*	0.27	0.6	0.002	0.97	5.68	0.02*
(2)×(3)	3	4.11	0.007*	0.2	0.89	2.68	0.05*	1.97	0.12	1.5	0.22
(1)×(4)	1	0.17	0.68	10.68	0.001*	0.37	0.54	1.71	0.19	15.7	0.0001*
(2)×(4)	3	0.08	0.97	4.95	0.002*	4.13	0.007*	5.39	0.001*	3.92	0.009*
(3)×(4)	1	3.52	0.06	5.98	0.02*	0.02	0.89	3.09	0.08	7.2	0.008*
(1)×(2)×(3)	3	0.35	0.79	2.99	0.03*	6.5	0.0003*	6.74	0.0002*	4.06	0.008*
(1)×(2)×(4)	3	0.69	0.55	0.35	0.79	2.45	0.06	0.66	0.57	1.06	0.37
(1)×(3)×(4)	1	7.21	0.008*	3.45	0.06	0.32	0.57	0.004	0.94	11.33	0.0009*
(2)×(3)×(4)	3	8.69	<0.0001*	1.38	0.25	1.27	0.29	0.58	0.63	4.76	0.003*
(1)×(2)×(3)×(4)	3	4.2	0.007*	4.08	0.008*	0.58	0.63	0.63	0.59	6.65	0.0003*
residuals	191										

* significant effect. Level of significance is α = 5%.

#### Effect of LbFV

**Figure 2:**
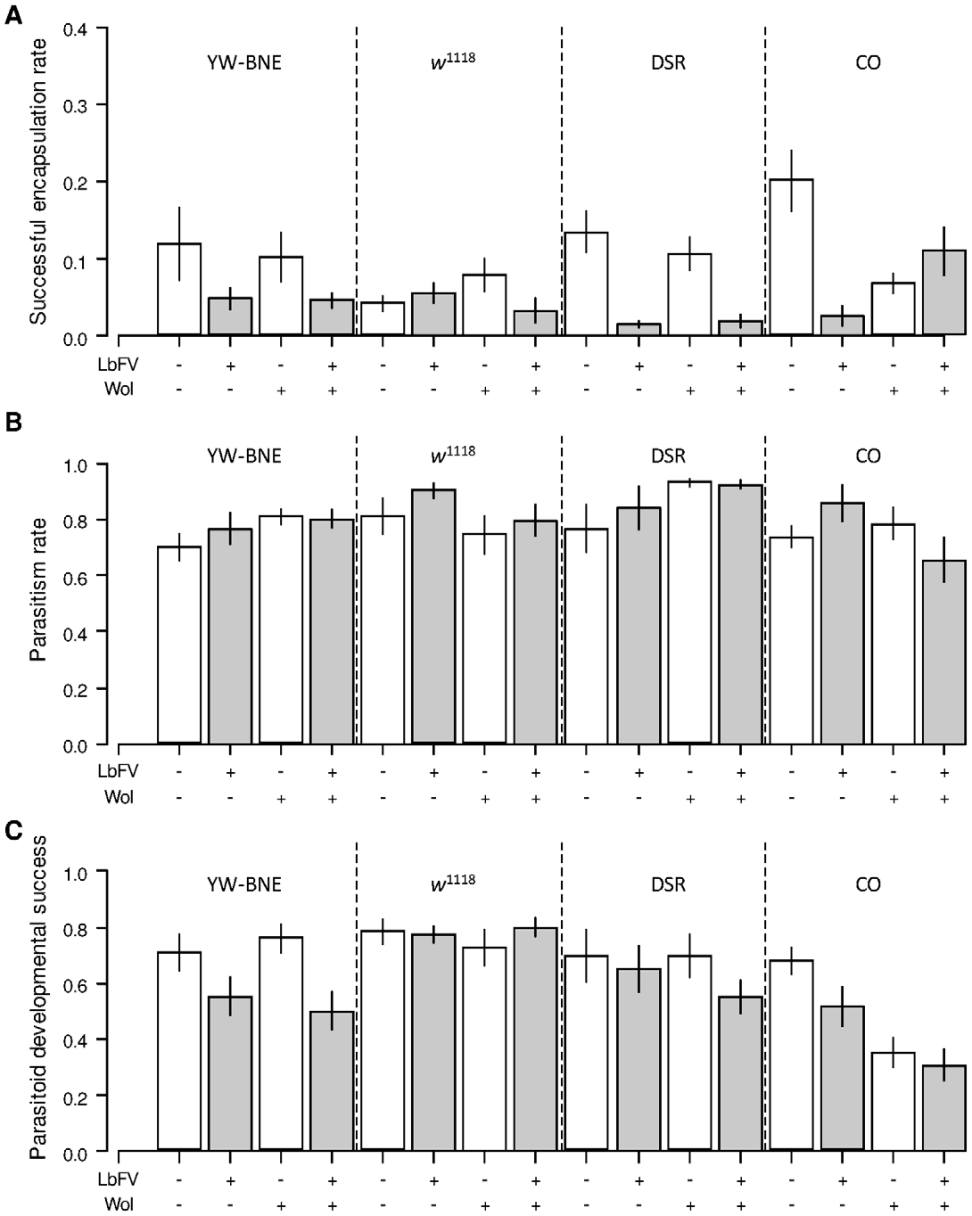
Fitness-related traits in experiment 1. (A) Successful encapsulation rate; (B) Parasitism rate; (C) Parasitoid developmental success ; (−) not infected; (+) infected; (Wol) *Wolbachia*. White and grey: virus-free and virus-infected parasitoids respectively. Bars are standard errors.

Successful encapsulation rates were relatively low: on average 7.5% of parasitized fly larvae successfully encapsulated the parasitoid(s) and survived to the adult stage (Figure 2A). Despite this low range in encapsulation level, significant differences were found according to the virus infection status. Virus-infected parasitoids were less frequently encapsulated than their uninfected counterparts (4.4% versus 10.6%; Figure 2A; Table 2). Analysis of the data per *Drosophila* nuclear background indicated that this difference was significant in both *D. simulans* nuclear backgrounds (DSR: *F_1,41_* = 47.52, *P*<0.0001; CO: *F_1,48_* = 13.26, *P* = 0.0007; Table S1). In *D. melanogaster* nuclear backgrounds, a similar trend was observed but was only marginally significant when corrected for multiple comparisons (Level of significance: 0.0125; YW-BNE: *F_1,52_* = 5.3, *P* = 0.03; *w* ^1118^: *F_1,50_* = 2.98, *P* = 0.09; Table S1). Importantly, the virus effect was independent of the block effect (Table 2, Table S1).

Virus-infected parasitoids tended to show a slightly higher parasitism rate but this difference was not significant (Figure 2B ; Table 2). The viral infection had, on average, a negative effect on parasitoid developmental success (Figure 2C ; Table 2), even if the decrease was only significant in YW-BNE flies (*F_1,52_* = 12.98; *P*<0.0007; Table S1).

**Figure 3:**
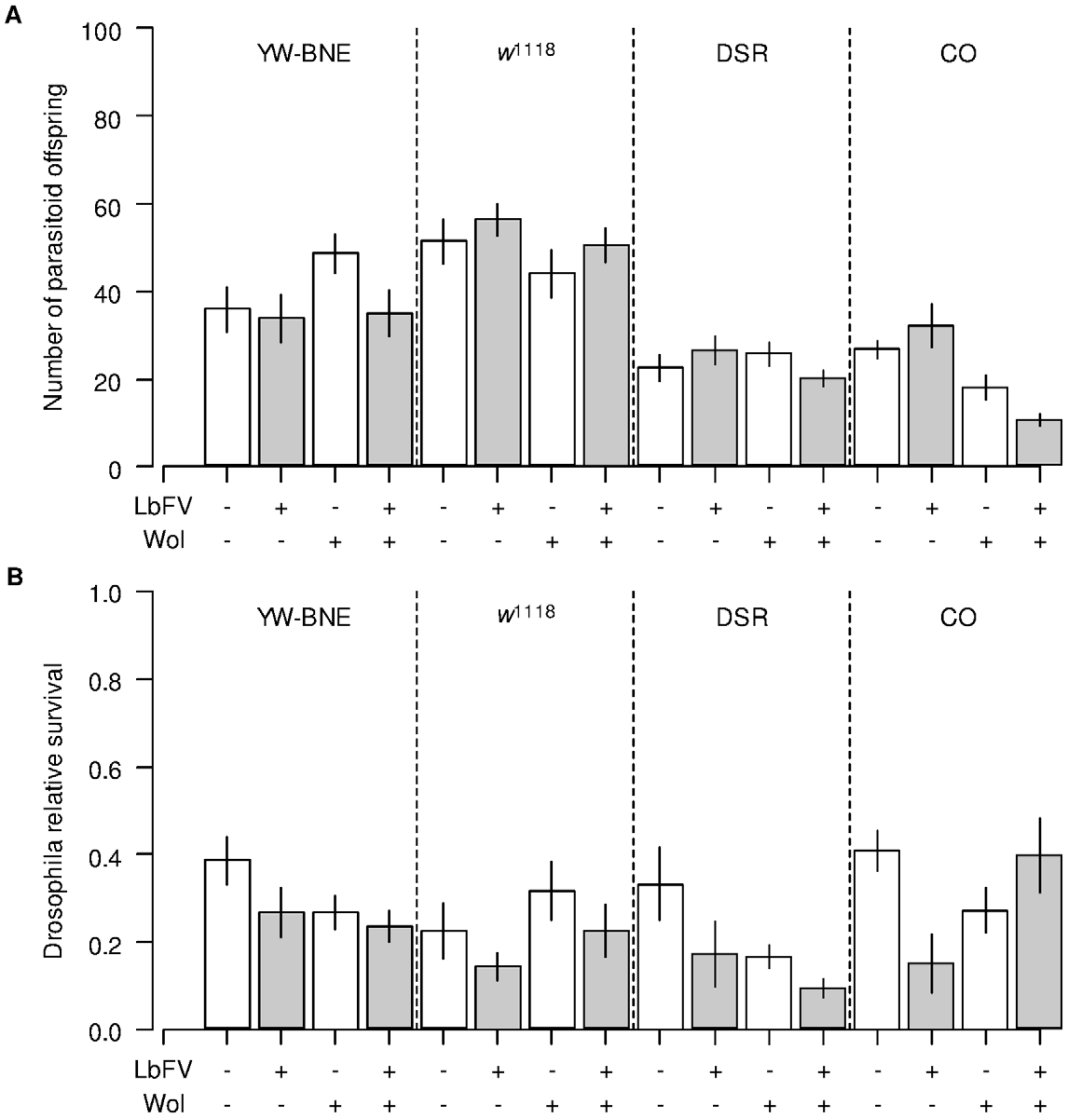
Overall fitness of parasitoids and *Drosophila* hosts in experiment 1. (A) Number of parasitoid offspring ; (B) *Drosophila* relative survival; (−) not infected; (+) infected; (Wol) *Wolbachia*. White and grey: virus-free and virus-infected parasitoids respectively. Bars are standard errors.

Both virus-infected and virus-free parasitoids produced a similar number of offspring (Figure 3A ; Table 2). On the host side, the presence of the virus decreased *Drosophila* relative survival, consistent with the lower successful encapsulation rate of virus-infected parasitoids (Figure 3B ; Table 2). This negative effect of the virus on *Drosophila* fitness was significant in both *D. simulans* nuclear backgrounds (DSR: *F_1,41_* = 9.15, *P* = 0.004; CO: *F_1,48_* = 21.52, *P*<0.0001; Table S1) and involved an interaction with the block effect for CO background. A similar trend, marginally significant, was observed in *D. melanogaster* (YW-BNE: *F_1,52_* = 3.95, *P* = 0.052; *w* ^1118^: *F_1,50_* = 3.83, *P* = 0.06; Table S1).

#### Effect of *Wolbachia*

Overall, *Wolbachia* did not impact the ability of flies to escape parasitism (no effect on parasitism rate), or their ability to successfully encapsulate parasitoids (Figure 2A; Table 2). However, *Wolbachia* presence correlated with a slight reduction in parasitoid developmental success (Figure 2C; Table 2) but this effect was only significant in CO flies (*F_1,48_* = 15.62; *P* = 0.0003; Table S1). Consistently, there was a tendency for a decrease in the number of parasitoids in the presence of *Wolbachia* (Table 2) but this effect was again only significant in CO flies (*F_1,48_* = 17.08; *P*<0.0001; Table S1).

#### LbFV-by-*Wolbachia* interaction

There was a marginally significant LbFV-by-*Wolbachia* interaction for successful encapsulation rate and significant LbFV-by-*Wolbachia* interactions for parasitism rate as well as for *Drosophila* relative survival (Figure 2A, 2B & 3B; Table 2). However, the analysis per nuclear background revealed that these virus-by-*Wolbachia* interactions for these traits were only significant within CO background (Successful encapsulation rate: *F_1,48_* = 23.93, *P*<0.0001; Parasitism rate: *F_1,48_* = 7.68, *P* = 0.008; *Drosophila* relative survival: *F_1,48_* = 26.5; *P*<0.0001; Table S1).

Within this background, *Wolbachia* (*w*Au) infection was correlated with a reduction in the successful encapsulation rate of virus-free parasitoids (Tukey's honest significance test, *P* = 0.01), but with a slight increase in the encapsulation rate of virus-infected parasitoids (Tukey's honest significance test, *P* = 0.02). Also, within this background, *Wolbachia* (*w*Au) infection was correlated with a significant reduction in parasitism rate when *Drosophila* were attacked by infected parasitoids (Tukey's honest significance test, *P* = 0.03) but was not correlated with parasitism rate when *Drosophila* were attacked by uninfected parasitoids (Tukey's honest significance test, *P* = 0.91). Finally, *Wolbachia* (*w*Au) infection had no effect on *Drosophila* relative survival when CO flies were exposed to virus-free parasitoids (Tukey's honest significance test, *P* = 0.61) whereas *w*Au-free flies had a lower survival than *w*Au-infected flies when exposed to virus-infected parasitoids (Tukey's honest significance test, *P* = 0.001).

We must stress that these virus-by-*Wolbachia* interactions should be interpreted with care since they were all highly dependent on the temporal block, according to the significant interactions of third order (Successful encapsulation rate: *F_1,48_* = 17.78; *P*<0.0001; Parasitism rate: *F_1,48_* = 39.64; *P*<0.0001; *Drosophila* relative survival: *F_1,48_* = 11.44; *P*<0.001; Table S1).

### Experiment 2: Direct and indirect effects of LbFV on encapsulation

**Figure 4:**
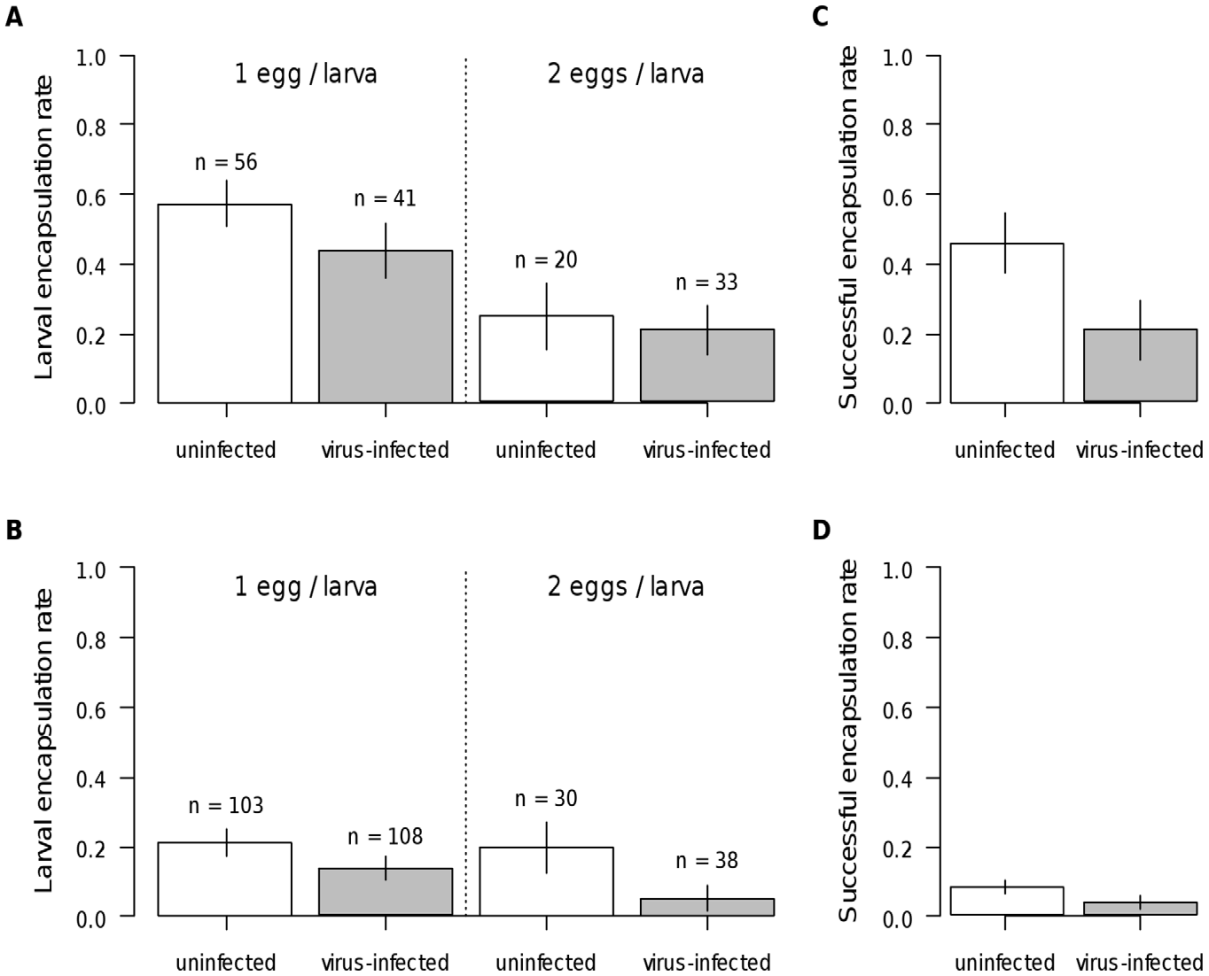
Encapsulation rates in cured DSR flies in experiment 2. Top: experiment 2.1 (low larval density). Bottom: experiment 2.2 (high larval density). (A & B) Larval encapsulation rate. (C & D) Successful encapsulation rate with “n” giving the number of dissected larvae. White and grey: virus-free and virus-infected parasitoids respectively. Bars are standard errors.

The most important effect detected in experiment 1 was the decrease in encapsulation rate when parasitoids were infected by LbFV. As the virus modifies the way females distribute their eggs among *Drosophila* larvae, we tried to separate out a direct effect of the virus from a potential indirect effects of superparasitism on encapsulation. To this end, we used the *Wolbachia*-cured DSR *Drosophila* line in a second experiment since, in this line, the virus effect previously observed was strong. In this second experiment, measures on adult flies confirmed the result from experiment 1: virus-infected parasitoids are less often successfully encapsulated than virus-free parasitoids (Figure 4C & D; *F_1,75_* = 15.3; *P*<0.0001). There was also a high variability between experiments 2.1 (low larval density) and 2.2 (high larval density) with a significantly lower successful encapsulation rate in experiment 2.2 (*F_1,75_* = 38.18; *P*<0.0001).

In both experiment 2.1 and 2.2, substantial superparasitism rates (proportion of superparasitized *Drosophila* larvae among parasitized ones) were observed with both virus-infected (≈61% and 30% for low and high larval density respectively) and uninfected parasitoids (≈30% and 26% for low and high larval density respectively).

The dissections of larvae confirmed the previous finding obtained on adults that infected parasitoids were less frequently encapsulated than uninfected parasitoids (Figure 4A & B; Table 3). This effect involved both a direct and an indirect effect of the virus. Analysis of monoparasitized *Drosophila* larvae demonstrated a direct effect of the virus: LbFV-infected parasitoids had a 11.8% reduction in the chance of being encapsulated compared with uninfected parasitoids (*Dev* = 5.34; df = 1; *P* = 0.02). Additionally, superparasitized larvae showed a 11.7% decrease in successful encapsulation events (encapsulation of all developing parasitoids) compared with monoparasitized larvae (Figure 4A &B; Table 3). Since LbFV is associated with an increase in the superparasitism tendency of females, this effect constitutes an indirect effect of the virus on encapsulation.

**Table 3:** Analysis of the larval encapsulation rate in cured DSR flies in experiments 2.1 (low larval density) and 2.2 (high larval density).

	df	Deviance	*P*
Experiment	1	31.99	<0.0001*
Virus	1	7.99	0.005*
Superparasitism	1	9.74	0.002*
Experiment×virus	1	0.18	0.67
Experiment×superparasitism	1	1.77	0.18
Virus×superparasitism	1	0.18	0.67
Experiment×virus×superparasitism	1	1.18	0.28

* significant effect in the generalized linear model. Level of significance is α = 5%.

## Discussion

*Drosophila* hosts can suffer high mortality rates due to parasitoid attacks [33], [40]. As a consequence, resistance against parasitoids should be strongly selected for, and encapsulation is one very common host defense strategy [41]. The expression of resistance is however affected by various factors such as host genotype-by-parasitoid genotype interactions [42], [43] or trade-offs with other traits [44]. The influence of bacterial symbionts on encapsulation was only recently investigated [12], [18]. Here, we tested the effect of two symbionts on the outcome of the interaction between *Drosophila* of several nuclear backgrounds and the parasitoid *Leptopilina boulardi*. We demonstrated that the behavior-manipulating virus LbFV of the wasp can interplay with the immune reaction of the *Drosophila* host by increasing the virulence of the parasitoid. Additionally, the *Wolbachia* strain *w*Au affected the encapsulation rate in CO flies, however, the direction of this effect depended on the parasitoid's infection status and it was not observed with any of the other *Wolbachia* strains tested.

In a first experiment, we tested the effect of LbFV and *Wolbachia* across different *Drosophila* nuclear backgrounds. *Drosophila* parasitized by virus-infected parasitoids had a lower successful encapsulation rate. This trend was consistent for all four *Drosophila* nuclear backgrounds tested but only significant in the *D. simulans* CO and DSR backgrounds. The non-significant trend in YW-BNE and *w* ^1118^ possibly results from a low statistical power due to the overall low encapsulation rate rather than a true absence of virus effect.

In a second set of experiments using *Wolbachia*-cured DSR flies as hosts, we tested whether this virus effect is caused by a direct effect on encapsulation or by an indirect effect of the increased tendency to superparasitize of virus-infected females. Considering that encapsulation is a costly physiological process, we should expect that flies would not be able to encapsulate more than a few parasitoids. Thus, the higher the superparasitism rate is, the lower the encapsulation rate should be. Dissections of larvae showed that the successful encapsulation rate variation measured on adult flies was indeed partly explained by the occurrence of superparasitism. Superparasitized larvae often failed to encapsulate all parasitoids whereas monoparasitized larvae succeeded more frequently, a result that is consistent with earlier studies on other host-parasitoid systems [45], [46]. For instance, in *Spodoptera littoralis* exposed to superparasitism by *Microplitis rufiventris*, a decrease in both cellular (encapsulation) and humoral response efficiencies was demonstrated [45].

In addition to this indirect effect of the virus on encapsulation rate through the induction of superparasitism, we also demonstrated a significant direct effect of the virus. In monoparasitized larvae, the presence of LbFV was associated with a decrease in larval encapsulation rate. This effect may arise either because the *Drosophila* immune response is depressed by the presence of the virus, or because infected-parasitoids have an increased virulence ability. The mechanism responsible for this protection, yet unknown, could involve either a virus-driven immune suppression as observed with polydnaviruses [47] or an evasion of the immune system [48], [49].

Whereas the direct protective effect of the virus is clearly advantageous for the parasitoid, the fitness reward from the indirect effect of superparasitism is unclear. In our experiment, one single female was put in each treatment vial, and could therefore directly benefit from self-superparasitism. In nature, however, conspecific-superparasitism is likely to be much more frequent than self-superparasitism. In such conditions, it is unknown if the superparasitizing female would benefit from the protective effect offered by superparasitism since this would depend on the outcome of the within-*Drosophila* competition between parasitoid larvae.

Besides encapsulation, the virus also negatively affected parasitoid developmental success. This virus effect could be either a direct effect of the physiological cost of infection, or an indirect effect of the increased superparasitism in infected parasitoids, as suggested by a previous study [50]. Indeed, virus-infected parasitoids are expected to develop more frequently in superparasitized larvae and must cope with intense competition. Despite this cost on developmental success, virus-infected and uninfected parasitoids produced similar numbers of offspring in all tested host-parasitoid combinations suggesting that this cost is compensated by the virus-mediated decrease in successful encapsulation.

Except for *w*Au, *Wolbachia* did not affect any of the tested traits. A positive effect of *Wolbachia* on the successful encapsulation rate was expected, at least for *w*Mel and *w*MelPop since these strains have previously been shown to increase hemolymph melanization, a key reaction involved in encapsulation, in both *D. melanogaster* and *D. simulans* [51]. No effect was however detected for *w*Mel, *w*MelPop nor *w*Ri. Fytrou *et al.* (2006) found that *w*Ri-infected DSR flies were less efficient in encapsulating the parasitoid *Leptopilina heterotoma*. Their results differ from our findings on the similar DSR *Drosophila* line, and indicate that the final outcome of host-parasitoid interactions also depends on the parasitoid species.

A surprising result was the complex interaction observed between viral infection in the parasitoid and infection by *Wolbachia* in *Drosophila* that was only observed with the strain *w*Au in *D. melanogaster*. Overall, *Wolbachia*-free CO flies suffered more from virus-infected parasitoid attacks than *w*Au-infected flies did. The slight increase in the encapsulation rate of virus-infected parasitoids suggests that a *w*Au-mediated protection might be activated in presence of LbFV. This is consistent with the strong antiviral protection of *w*Au in CO flies, allowing resistance against the RNA virus DCV [23]. However, the effect on encapsulation was not detected for the other *Wolbachia* strains tested, although they were also found to have an antiviral activity in previous studies [6], [22], [23]. In addition, virus-infected parasitoids exhibited higher parasitism rates on *Wolbachia*-free than on *Wolbachia*-infected larvae, whereas virus-free parasitoids displayed a similar parasitism rate whatever the infection status of CO flies. This result suggests that *w*Au might either influence the ability of infected parasitoids to locate *Drosophila* larvae, modify their egg-laying preferences or that *w*Au-infected *Drosophila* larvae might be better at avoiding parasitoid attacks when the parasitoid is infected by the virus. We must however be cautious as all these interaction effects strongly depended on the temporal block and thus on unknown environmental parameters. Further investigations should be carried out before concluding that *w*Au can be beneficial to its host, and to determine by which way *w*Au interacts with LbFV.

In conclusion, our data confirm that symbionts in hosts and parasitoids contribute to variation in extremely important phenotypes such as resistance and virulence, in addition to classical nuclear factors [42], [52]. Results also encourage a reconsideration of the cost-benefit balance of LbFV infection for *L. boulardi*. A virus-induced increase in *L. boulardi*'s virulence might depict an ongoing evolution towards a mutualistic association between the virus and the parasitoid, similar to what is believed to have occurred between ancestral polydnaviruses and their wasp carriers [15]. From the host side, we again demonstrated, but only for *w*Au strain, that *Wolbachia* might not only be a reproductive parasite in arthropods, but may as well contribute to variation of traits involved in host-parasitoid interactions. Because symbionts benefit from vertical transmission, they produce heritable variation on which natural selection can act and directly contribute to the adaptation of their host. As such, there is a crucial need to view infections by so-called parasites in a broader ecological context by considering several life-history traits of their hosts and their interactions with other species within the community [53]. More generally, we should also take symbionts into account as a potential force shaping this community [54].

## Supporting Information

Table S1 **Analysis of variance of life-history per** ***Drosophila*** **nuclear background in experiment 1.** * Significant effect; Level of significance: α = 0.0125 (Bonferroni correction for multiple comparisons). Successful encapsulation rate: square root-transformed data; Parasitism rate: arcsine square root-transformed data; Parasitoid developmental success: square root-transformed data; Number of parasitoid offspring: square root-transformed data; Drosophila relative survival: log-transformed data.
(DOC)

## References

[1] Moran N (2007). Symbiosis as an adaptive process and source of phenotypic complexity.. Proc Natl Acad Sci USA.

[2] Gilbert SF, McDonald E, Boyle N, Buttino N, Gyi L (2010). Symbiosis as a source of selectable epigenetic variation: taking the heat for the big guy.. Philos T Roy Soc B.

[3] Scarborough CL, Ferrari J, Godfray HCJ (2005). Aphid protected from pathogen by endosymbiont.. Science.

[4] Oliver K, Russell J, Moran N, Hunter M (2003). Facultative bacterial symbionts in aphids confer resistance to parasitic wasps.. Proc Natl Acad Sci USA.

[5] Goodrich-Blair H, Clarke DJ (2007). Mutualism and pathogenesis in *Xenorhabdus* and *Photorhabdus*: two roads to the same destination.. Mol Microbiol.

[6] Teixeira L, Ferreira A, Ashburner M (2008). The Bacterial Symbiont *Wolbachia* Induces Resistance to RNA Viral Infections in *Drosophila melanogaster*.. Plos Biol.

[7] Jaenike J, Unckless R, Cockburn SN, Boelio LM, Perlman SJ (2010). Adaptation via Symbiosis: Recent Spread of a *Drosophila* Defensive Symbiont.. Science.

[8] Schneider DS, Chambers MC (2008). Rogue Insect Immunity.. Science.

[9] Oliver KM, Degnan PH, Burke GR, Moran NA (2010). Facultative Symbionts in Aphids and the Horizontal Transfer of Ecologically Important Traits.. Annu Rev Entomol.

[10] Vorburger C, Sandrock C, Gouskov A, Castaneda LE, Ferrari J (2009). Genotypic variation and the role of defensive endosymbionts in an all-parthenogenetic host-parasitoid interaction.. Evolution.

[11] Vorburger C, Gehrer L, Rodriguez P (2010). A strain of the bacterial symbiont *Regiella insecticola* protects aphids against parasitoids.. Biol Letters.

[12] Xie JL, Vilchez I, Mateos M (2010). *Spiroplasma* Bacteria Enhance Survival of *Drosophila hydei* Attacked by the Parasitic Wasp *Leptopilina heterotoma*.. Plos One.

[13] Stasiak K, Renault S, Federici BA, Bigot Y (2005). Characteristics of pathogenic and mutualistic relationships of ascoviruses in field populations of parasitoid wasps.. J Insect Physiol.

[14] Renault S, Stasiak K, Federici B, Bigot Y (2005). Commensal and mutualistic relationships of reoviruses with their parasitoid wasp hosts.. J Insect Physiol.

[15] Bezier A, Annaheim M, Herbiniere J, Wetterwald C, Gyapay G (2009). Polydnaviruses of Braconid Wasps Derive from an Ancestral Nudivirus.. Science.

[16] Volkoff AN, Jouan V, Urbach S, Samain S, Bergoin M (2010). Analysis of Virion Structural Components Reveals Vestiges of the Ancestral Ichnovirus Genome.. Plos Pathog.

[17] Drezen JM, Bézier A, Lesobre J, Huguet E, Dupuy C (2006). Virulence genes of parasitoid wasps encoded by symbiotic viruses. ICOPA XI: Proceedings of the 11th International Congress of Parasitology.

[18] Fytrou A, Schofield PG, Kraaijeveld AR, Hubbard SF (2006). *Wolbachia* infection suppresses both host defence and parasitoid counter-defence.. P Roy Soc Lond B Bio.

[19] Carton Y, Nappi AJ (1997). *Drosophila* cellular immunity against parasitoids.. Parasitol Today.

[20] Hoffmann AA, Clancy D, Duncan J (1996). Naturally-occurring *Wolbachia* infection in *Drosophila simulans* that does not cause cytoplasmic incompatibility.. Heredity.

[21] Merçot H, Charlat S (2004). *Wolbachia* infections in *Drosophila melanogaster* and *D. simulans*: polymorphism and levels of cytoplasmic incompatibility.. Genetica.

[22] Hedges L, Brownlie J, O'Neill S, Johnson K (2008). *Wolbachia* and Virus Protection in Insects.. Science.

[23] Osborne SE, Leong YS, O'Neill SL, Johnson KN (2009). Variation in Antiviral Protection Mediated by Different *Wolbachia* Strains in *Drosophila simulans*.. Plos Pathog.

[24] Frentiu FD, Robinson J, Young PR, McGraw EA, O'Neill SL (2010). *Wolbachia*-Mediated Resistance to Dengue Virus Infection and Death at the Cellular Level.. Plos One.

[25] Walker T, Johnson PH, Moreira LA, Iturbe-Ormaetxe I, Frentiu FD (2011). The *w*Mel *Wolbachia* strain blocks dengue and invades caged *Aedes aegypti* populations.. Nature.

[26] Moreira LA, Iturbe-Ormaetxe I, Jeffery JA, Lu GJ, Pyke AT (2009). A *Wolbachia* Symbiont in *Aedes aegypti* Limits Infection with Dengue, Chikungunya, and *Plasmodium*.. Cell.

[27] Kambris Z, Cook PE, Phuc HK, Sinkins SP (2009). Immune Activation by Life-Shortening *Wolbachia* and Reduced Filarial Competence in Mosquitoes.. Science.

[28] Hughes GL, Koga R, Xue P, Fukatsu T, Rasgon JL (2011). *Wolbachia* Infections Are Virulent and Inhibit the Human Malaria Parasite *Plasmodium Falciparum* in *Anopheles Gambiae*.. Plos Pathog.

[29] Glaser RL, Meola MA (2010). The Native *Wolbachia* Endosymbionts of *Drosophila melanogaster* and *Culex quinquefasciatus* Increase Host Resistance to West Nile Virus Infection.. Plos One.

[30] Chevalier F, Herbinière-Gaboreau J, Bertaux J, Raimond M, Morel F (2010). The Immune Cellular Effectors of Terrestrial Isopod *Armadillidium vulgare*: Meeting with Their Invaders, *Wolbachia*.. Plos One.

[31] Sicard M, Chevalier F, De Vlechouver M, Bouchon D, Greve P (2010). Variations of immune parameters in terrestrial isopods: a matter of gender, aging and *Wolbachia*.. Naturwissenschaften.

[32] Cerenius L, Soderhall K (2004). The prophenoloxidase-activating system in invertebrates.. Immunol Rev.

[33] Patot S, Martinez J, Allemand R, Gandon S, Varaldi J (2010). Prevalence of a virus inducing behavioural manipulation near species range border.. Mol Ecol.

[34] Varaldi J, Fouillet P, Ravallec M, Lopez-Ferber M, Boulétreau M (2003). Infectious Behavior in a Parasitoid.. Science.

[35] Gandon S, Rivero A, Varaldi J (2006). Superparasitism evolution: Adaptation or manipulation?. Am Nat.

[36] David J (1962). A new medium for rearing *Drosophila* in axenic condition.. Drosophila Information Service.

[37] Zhou WG, Rousset F, O'Neill S (1998). Phylogeny and PCR-based classification of *Wolbachia* strains using wsp gene sequences.. P Roy Soc Lond B Bio.

[38] Varaldi J, Ravallec M, Labrosse C, Lopez-Ferber M, Boulétreau M (2006). Artifical transfer and morphological description of virus particles associated with superparasitism behaviour in a parasitoid wasp.. J Insect Physiol.

[39] Patot S, Lepetit D, Charif D, Varaldi J, Fleury F (2009). Molecular Detection, Penetrance, and Transmission of an Inherited Virus Responsible for Behavioral Manipulation of an Insect Parasitoid.. Appl Environ Microb.

[40] Fleury F, Ris N, Allemand R, Fouillet P, Carton Y (2004). Ecological and genetic interactions in *Drosophila*–parasitoids communities: a case study with *D. melanogaster*, *D. simulans* and their common *Leptopilina* parasitoids in south-eastern France.. Genetica.

[41] Carton Y, Nappi AJ (2001). Immunogenetic aspects of the cellular immune response of *Drosophila* against parasitoids.. Immunogenetics.

[42] Dupas S, Carton Y, Poirie M (2003). Genetic dimension of the coevolution of virulence-resistance in *Drosophila* - parasitoid wasp relationships.. Heredity.

[43] Dubuffet A, Dupas S, Frey F, Drezen JM, Poirié M (2007). Genetic interactions between the parasitoid wasp *Leptopilina boulardi* and its *Drosophila* hosts.. Heredity.

[44] Kraaijeveld AR, Limentani EC, Godfray HCJ (2001). Basis of the trade-off between parasitoid resistance and larval competitive ability in *Drosophila melanogaster*.. P Royal Soc Lond B Bio.

[45] Hegazi E, Khafagi W (2008). The effects of host age and superparasitism by the parasitoid, *Microplitis rufiventris* on the cellular and humoral immune response of *Spodoptera littoralis* larvae.. J Invertebr Pathol.

[46] Giordanengo P, Nenon JP (1990). Melanization and encapsulation of eggs and larvae of *Epidinocarsis lopezi* by its host *Phenacoccus manihoti* - Effects of superparasitism and egg-laying patterns.. Entomol Exp Appl.

[47] Beckage NE (1998). Modulation of immune responses to parasitoids by polydnaviruses.. Parasitology.

[48] Asgari S, Theopold U, Wellby C, Schmidt O (1998). A protein with protective properties against the cellular defense reactions in insects.. Proc Natl Acad Sci USA.

[49] Kinuthia W, Li DM, Schmidt O, Theopold U (1999). Is the surface of endoparasitic wasp eggs and larvae covered by a limited coagulation reaction?. J Insect Physiol.

[50] Varaldi J, Boulétreau M, Fleury F (2005). Cost induced by viral particles manipulating superparasitism behaviour in the parasitoid *Leptopilina boulardi*.. Parasitology.

[51] Thomas P, Kenny N, Eyles D, Moreira LA, O'Neill SL (2011). Infection with the *w*Mel and *w*MelPop strains of *Wolbachia* leads to higher levels of melanization in the hemolymph of *Drosophila melanogaster*, *Drosophila simulans* and *Aedes aegypti*.. Dev Comp Immunol.

[52] Dupas S, Frey F, Carton Y (1998). A single parasitoid segregating factor controls immune suppression in *Drosophila*.. J Hered.

[53] Sternberg ED, Lefèvre T, Rawstern AH, de Roode JC (2011). A virulent parasite can provide protection against a lethal parasitoid.. Infection, Genetics and Evolution.

[54] Ferrari J, Vavre F (2011). Bacterial symbionts in insects or the story of communities affecting communities.. Philos T Roy Soc B.

[55] Yamada R, Floate KD, Riegler M, O'Nein SL (2007). Male development time influences the strength of *Wolbachia*-Induced cytoplasmic incompatibility expression in *Drosophila melanogaster*.. Genetics.

[56] Min KT, Benzer S (1997). *Wolbachia*, normally a symbiont of *Drosophila*, can be virulent, causing degeneration and early death.. Proc Natl Acad Sci USA.

[57] Hoffmann AA, Turelli M, Simmons GM (1986). Unidirectional incompatibility between populations of *Drosophila simulans*.. Evolution.

