# Supplementary material for: Influence of the Virus LbFV and of Wolbachia in a Host-Parasitoid Interaction
Source: PLoS One. 2012 Apr 25;7(4):e35081. doi: 10.1371/journal.pone.0035081 (PMC3338833; doi:10.1371/journal.pone.0035081)
Supplement: Table S1 — Analysis of variance of life-history per Drosophila nuclear background in experiment 1. * Significant effect; Level of significance: α = 0.0125 (Bonferroni correction for multiple comparisons). Successful encapsulation rate: square root-transformed data; Parasitism rate: arcsine square root-transformed data; Parasitoid developmental success: square root-transformed data; Number of parasitoid offspring: square root-transformed data; Drosophila relative survival: log-transformed data. (DOC) [file pone.0035081.s001.doc]

**Table S1. Analysis of variance of life-history per *Drosophila* nuclear background in experiment 1.**

| Drosophila nuclear background | Parameters |  | Successful encapsulation rate | | Parasitism rate | | Parasitoid developmental success | | Number of parasitoid offspring | | Drosophila relative survival | |
| --- | --- | --- | --- | --- | --- | --- | --- | --- | --- | --- | --- | --- |
|  |  | df | F | *P* | F | *P* | F | *P* | F | *P* | F | *P* |
| YW-BNE | Block (1) | 1 | 6.64 | 0.01* | 5.87 | 0.02 | 0.56 | 0.46 | 1.73 | 0.19 | 6.75 | 0.01* |
|  | Virus (2) | 1 | 5.3 | 0.03 | 0.79 | 0.38 | 12.98 | 0.0007* | 3.28 | 0.08 | 3.95 | 0.052 |
|  | Wolbachia (3) | 1 | 0.11 | 0.74 | 3.05 | 0.09 | 0.03 | 0.86 | 1.69 | 0.2 | 1.56 | 0.22 |
|  | interactions |  |  |  |  |  |  |  |  |  |  |  |
|  | (1) × (2) | 1 | 0.29 | 0.59 | 0.78 | 0.38 | 16.76 | 0.0001* | 12.06 | 0.001* | 1.59 | 0.21 |
|  | (1) × (3) | 1 | 0.04 | 0.85 | 3.9 | 0.05 | 0.83 | 0.37 | 2.5 | 0.12 | 3.66 | 0.06 |
|  | (2) × (3) | 1 | 0.12 | 0.73 | 0.89 | 0.35 | 1.16 | 0.29 | 1.52 | 0.22 | 0.65 | 0.42 |
|  | (1) × (2) × (3) | 1 | 0.36 | 0.55 | 1.43 | 0.24 | 0.07 | 0.79 | 0.01 | 0.91 | 1.96 | 0.17 |
|  | residuals | 52 |  |  |  |  |  |  |  |  |  |  |
| *w*^1118^ | Block (1) | 1 | 9.26 | 0.004* | 0.24 | 0.62 | 0.07 | 0.8 | 0.001 | 0.98 | 0.52 | 0.47 |
|  | Virus (2) | 1 | 2.98 | 0.09 | 2.22 | 0.14 | 0.84 | 0.37 | 2.09 | 0.15 | 3.83 | 0.06 |
|  | Wolbachia (3) | 1 | 0.11 | 0.74 | 3.04 | 0.09 | 0.23 | 0.63 | 2.07 | 0.16 | 1.88 | 0.18 |
|  | interactions |  |  |  |  |  |  |  |  |  |  |  |
|  | (1) × (2) | 1 | 0.87 | 0.35 | 6.85 | 0.01* | 0.06 | 0.8 | 4.22 | 0.05 | 5.33 | 0.03 |
|  | (1) × (3) | 1 | 0.58 | 0.45 | 0.93 | 0.34 | 0.13 | 0.72 | 0.03 | 0.86 | 0.79 | 0.38 |
|  | (2) × (3) | 1 | 5.48 | 0.02 | 0.06 | 0.81 | 0.88 | 0.35 | 0.11 | 0.75 | 0.27 | 0.61 |
|  | (1) × (2) × (3) | 1 | 0.98 | 0.33 | 1.51 | 0.22 | 0.5 | 0.48 | 1.04 | 0.31 | 0.13 | 0.72 |
|  | residuals | 50 |  |  |  |  |  |  |  |  |  |  |

| Drosophila nuclear background | Parameters |  | Successful encapsulation rate | | Parasitism rate | | Parasitoid developmental success | | Number of parasitoid offspring | | Drosophila relative survival | |
| --- | --- | --- | --- | --- | --- | --- | --- | --- | --- | --- | --- | --- |
|  |  | df | F | *P* | F | *P* | F | *P* | F | *P* | F | *P* |
| CO | Block (1) | 1 | 0.21 | 0.65 | 0.17 | 0.69 | 6.21 | 0.02 | 2.2 | 0.14 | 4.14 | 0.05 |
|  | Virus (2) | 1 | 13.26 | 0.0007* | 0.58 | 0.45 | 2.65 | 0.11 | 0.65 | 0.42 | 21.52 | < 0.0001* |
|  | Wolbachia (3) | 1 | 0.15 | 0.7 | 2.81 | 0.1 | 15.62 | 0.0003* | 17.08 | 0.0001* | 6.35 | 0.01* |
|  | interactions |  |  |  |  |  |  |  |  |  |  |  |
|  | (1) × (2) | 1 | 0.11 | 0.74 | 3.74 | 0.06 | 0.1 | 0.75 | 0.72 | 0.4 | 12.78 | 0.0008* |
|  | (1) × (3) | 1 | 1.28 | 0.26 | 5.33 | 0.03 | 0.34 | 0.56 | 0.48 | 0.49 | 14.37 | 0.0004* |
|  | (2) × (3) | 1 | 23.93 | < 0.0001* | 7.68 | 0.008* | 1.95 | 0.17 | 1.4 | 0.24 | 26.5 | < 0.0001* |
|  | (1) × (2) × (3) | 1 | 17.78 | 0.0001* | 11.44 | 0.001* | 1.08 | 0.3 | 1.05 | 0.31 | 39.64 | < 0.0001* |
|  | residuals | 48 |  |  |  |  |  |  |  |  |  |  |
| DSR | Block (1) | 1 | 2.32 | 0.14 | 0.69 | 0.41 | 0.02 | 0.9 | 0.03 | 0.87 | 2.02 | 0.16 |
|  | Virus (2) | 1 | 47.52 | < 0.0001* | 0.39 | 0.54 | 0.93 | 0.34 | 0.11 | 0.74 | 9.15 | 0.004* |
|  | Wolbachia (3) | 1 | 1.02 | 0.32 | 5.47 | 0.02 | 0.01 | 0.91 | 0.02 | 0.89 | 2.63 | 0.11 |
|  | interactions |  |  |  |  |  |  |  |  |  |  |  |
|  | (1) × (2) | 1 | 0.1 | 0.75 | 0.05 | 0.82 | 3.1 | 0.09 | 1.17 | 0.29 | 0.02 | 0.88 |
|  | (1) × (3) | 1 | 0.65 | 0.42 | 2.53 | 0.12 | 4.02 | 0.05 | 0.16 | 0.69 | 3.74 | 0.06 |
|  | (2) × (3) | 1 | 0.27 | 0.61 | 1.03 | 0.32 | 0.13 | 0.72 | 1.07 | 0.31 | 0.56 | 0.45 |
|  | (1) × (2) × (3) | 1 | 0.43 | 0.52 | 0.32 | 0.58 | 0.35 | 0.56 | 0.0009 | 0.98 | 0.24 | 0.63 |
|  | residuals | 41 |  |  |  |  |  |  |  |  |  |  |

* Significant effect ; Level of significance : α = 0.0125 (Bonferroni correction for multiple comparisons). Successful encapsulation rate : square root-transformed data ; Parasitism rate : arcsine square root-transformed data ; Parasitoid developmental success : square root-transformed data ; Number of parasitoid offspring : square root-transformed data ; Drosophila relative survival : log-transformed data.
